# Veterans Affairs patient database (VAPD 2014–2017): building nationwide granular data for clinical discovery

**DOI:** 10.1186/s12874-019-0740-x

**Published:** 2019-05-08

**Authors:** Xiao Qing Wang, Brenda M. Vincent, Wyndy L. Wiitala, Kaitlyn A. Luginbill, Elizabeth M. Viglianti, Hallie C. Prescott, Theodore J. Iwashyna

**Affiliations:** 1 Division of Pulmonary and Critical Care Medicine, Department of Internal Medicine, University of Michigan, 2800 Plymouth Road, Building 16, Floor 3, Ann Arbor, MI 48109 USA; 2 Center for Clinical Management Research, Veterans Affairs Ann Arbor Health System, 2800 Plymouth Road, Building 16, Floor 3, Ann Arbor, MI 48109 USA

**Keywords:** Laboratory values, Healthcare database, Patient physiology, Electronic health records, Hospitalization, sepsis

## Abstract

**Background:**

To study patient physiology throughout a period of acute hospitalization, we sought to create accessible, standardized nationwide data at the level of the individual patient-facility-day. This methodology paper summarizes the development, organization, and characteristics of the Veterans Affairs Patient Database 2014–2017 (VAPD 2014–2017). The VAPD 2014–2017 contains acute hospitalizations from all parts of the nationwide VA healthcare system with daily physiology including clinical data (labs, vitals, medications, risk scores, etc.), intensive care unit (ICU) indicators, facility, patient, and hospitalization characteristics.

**Methods:**

The VA data structure and database organization represents a complex multi-hospital system. We define a single-site hospitalization as one or more consecutive stays with an acute treating specialty at a single facility. The VAPD 2014–2017 is structured at the patient-facility-day level, where every patient-day in a hospital is a row with separate identification variables for facility, patient, and hospitalization. The VAPD 2014–2017 includes daily laboratory, vital signs, and inpatient medication. Such data were validated and verified through lab value range and comparison with patient charts. Sepsis, risk scores, and organ dysfunction definitions were standardized and calculated.

**Results:**

We identified 565,242 single-site hospitalizations (SSHs) in 2014; 558,060 SSHs in 2015; 553,961 SSHs in 2016; and 550,236 SSHs in 2017 at 141 VA hospitals. The average length of stay was four days for all study years. In-hospital mortality decreased from 2014 to 2017 (1.7 to 1.4%), 30-day readmission rates increased from 15.3% in 2014 to 15.6% in 2017; 30-day mortality also decreased from 4.4% in 2014 to 4.1% in 2017. From 2014 to 2017, there were 107,512 (4.8%) of SSHs that met the Center for Disease Control and Prevention’s Electronic Health Record-based retrospective definition of sepsis.

**Conclusion:**

The VAPD 2014–2017 represents a large, standardized collection of granular data from a heterogeneous nationwide healthcare system. It is also a direct resource for studying the evolution of inpatient physiology during both acute and critical illness.

**Electronic supplementary material:**

The online version of this article (10.1186/s12874-019-0740-x) contains supplementary material, which is available to authorized users.

## Background

As electronic health records (EHR) are now available in most U.S. hospitals, there is increasing interest in leveraging EHR data for research, performance measurement, and decision support. However, the process for recording clinical data varies by hospital, such that data extracted from individual hospitals must be standardized to create system-wide, patient-level databases.

The Veterans Affairs Patient Database 2014–2017 (VAPD 2014–2017) was created to contain daily physiological information for all patients hospitalized in 141 Veterans Affairs (VA) facilities. This is intended to facilitate the study of patient physiology throughout a period of hospitalization for acute illness, including before, during, and after an intensive care unit (ICU) stay. In addition, with many hospitals, it allows for study of hospital-level differences. The depth and breadth of the VAPD 2014–2017 sets it apart from other research datasets that contain physiological data only at hospital admission (e.g. Pennsylvania Health Care Cost Containment Council (PHC4) [1]), only during intensive care (e.g. Acute Physiology and Chronic Health Evaluation (APACHE) [2], Adult Patient Database (APD) [3], and Medical Information Mart for Intensive Care (MIMIC) [4]), or only in a single hospital (e.g. MIMIC).

In this paper, we present the development, organization, and characteristics of the VAPD 2014–2017. We describe the processes for extracting, verifying, and standardizing clinical data collected from 141 VA facilities. We then present patient and hospital characteristics.

This manuscript serves several purposes. It documents the development of the VAPD 2014–2017, and the decisions made during this process and thereby as a reference document for manuscripts using the VAPD 2014–2017; it serves as a guide for researchers wishing to standardize data across other multi-hospital systems; and it provides basic information about the data and patients in the VAPD 2014–2017, to help investigators determine whether the VAPD 2014–2017 would be an appropriate data source for answering particular research questions. All code required to extract and standardize the data as we did are also presented for use without copyright online [5].

### Construction and content

#### VA data structure

**Fig. 1:**
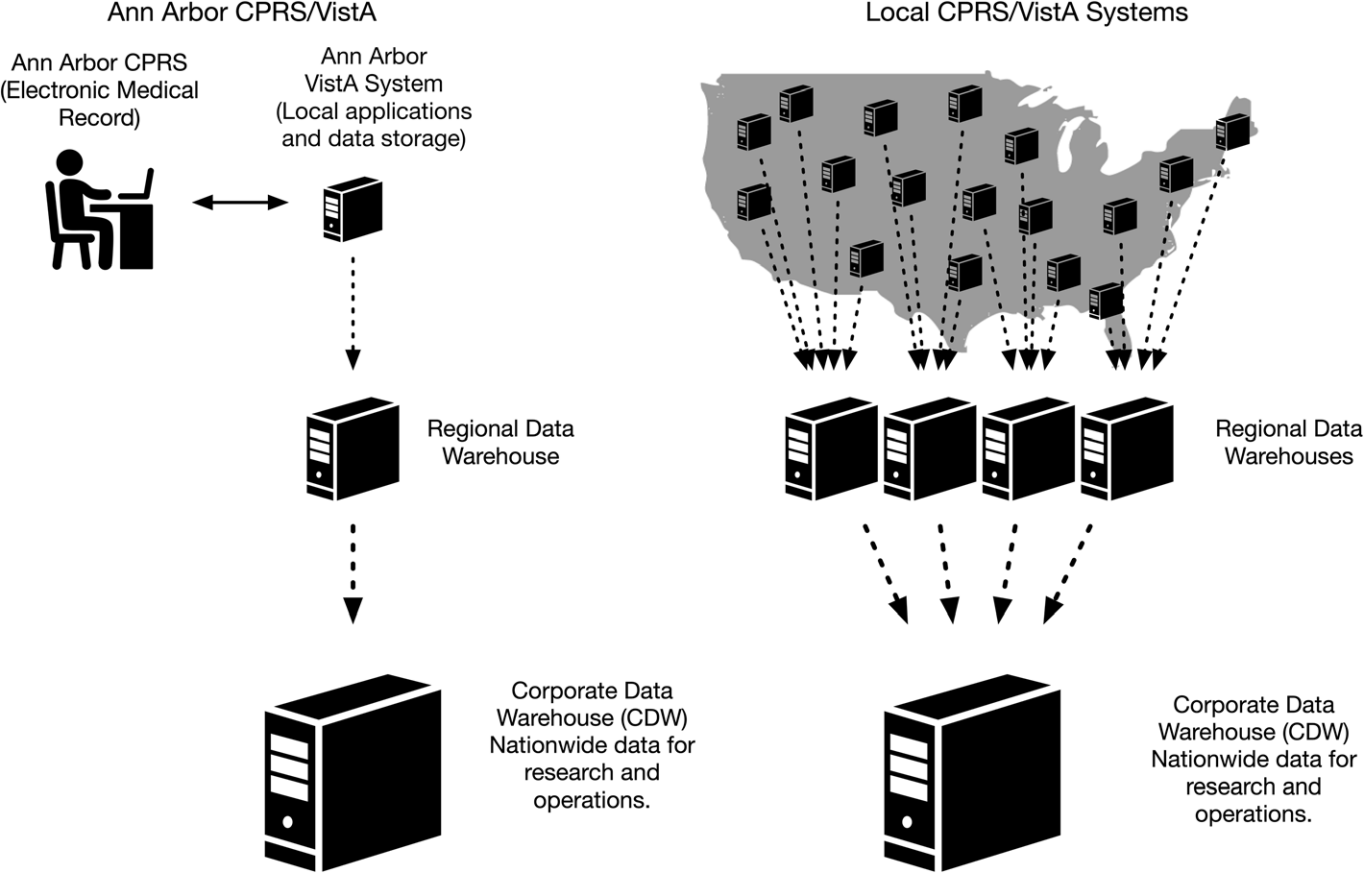
Data flow from local CPRS/VistA systems to the Corporate Data Warehouse (CDW). Legend: On the left, we depict the flow of data from a single local CPRS/VistA system (Ann Arbor), to a regional data warehouse, and ultimately, to the CDW. On the right, we depict the flow of data from local CPRS/VistA system, to four regional data warehouses, and ultimately to the CDW. (This Figure is intended to display the flow of data from around the country to the CDW, but does not depict the exact number and location of local CPRS/VistA systems). All figures are permitted for use without acknowledgement from The Noun Project (https://thenounproject.com/) of which we are members

Veterans Information Systems Technology Architecture (VistA) comprises over 180 clinical, financial and administrative applications that serve as the “back-end” to the VA’s electronic medical record, Computerized Patient Record System (CPRS) (Fig. 1) [6–8]. Since 2004, local CPRS/VistA systems have been used to document all clinical activities, orders, and results across the entire VA system. In one day, CPRS/VistA systems capture more than 1.2 million physician orders, 1 million vital signs, and 600,000 medication doses [7].

CPRS/VistA reflect the scope and magnitude of VA’s clinical activity nationwide [7, 9, 10]. In 2006, VA began collecting data from local CPRS/VistA systems around the country into a single central repository—the Corporate Data Warehouse (CDW) – the architecture of which has been previously described [7, 9, 10]. CDW collects 60 domains of data (e.g. demographics, laboratory results, medication orders, barcode medication administrations, vital signs, etc.) selected by clinical experts and operational leaders [7, 9, 10]. The CDW represents one of the largest clinical data warehouses, with over 10 million hospitalizations, 2.7 billion vital signs, and 6.6 billion laboratory results, and is updated several times each day with new data from the local CPRS/VistA systems [7, 9, 10].

#### Database organization

To construct the VAPD 2014–2017, we extracted data from the CDW for all acute hospitalizations between calendar years 2014 and 2017. The database is structured at the patient-facility-day level, so that every patient-day in a hospital is a row with separate identification variables to identify the hospitalization, the patient, and the hospital facility (Table 1). Patients may have more than one hospitalization, so may appear in the database multiple times, and are linkable on hospitalization and patient levels. For instance, when a patient was hospitalized at two different facilities on the same calendar day (e.g., the patient was transferred from one VA facility to another VA facility), the patient would have two rows in the database for that date, one for each facility.

**Table 1:** VAPD 2014–2017 standardized nomenclature

Term	Conceptual definition
Patient-facility-day	An individual calendar date that a patient spent in the hospital (sta6a)
Hospitalization	One or more consecutive acute specialty stays
Bedded stay	Any stay in a healthcare facility where a patient is provided a bed, including hospital, nursing facility, mental health facility, or domiciliary for homeless Veterans
Specialty stay	A portion of a bedded stay defined by the treating specialty. Each bedded stay is composed of one or more specialty stays
Specialty transfer	When patient’s care is transitioned from one treating specialty to another
Acute specialty stay	A specialty stay that is for an acute medical condition
Non-acute specialty stay	A specialty stay that is not for an acute medical condition
Facility laboratory code	Facility-specific code linked to lab test names
Facility LOINC	Facility-specific code linked to LOINC codes [12]
Facility laboratory test name	The name used to identify a lab test at a specific site (e.g. white blood cell count, WBC)
Laboratory test synonyms	Other clinical names for the same laboratory test (e.g. blood gas, carbon dioxide both map to the same lab test)
Topography	A specific description of an anatomic region of the body where lab specimen was drawn (e.g. arterial blood, plasma, blood, serum)

Terms are ordered by order of appearance in the text. A complete list of VAPD 2014–2017 standardized nomenclature can be found in Additional file 1: Appendix A of the Online Data Supplement

#### Defining hospitalizations

We used the methods of Vincent et al. [11] to define hospitalizations within VA. Briefly, VA data (specifically, the Specialty Transfer table in the Inpatient domain) are organized by the concept of bedded stay rather than the concept of a hospitalization (Table 1). A bedded stay is any stay in a healthcare facility where a patient is provided a bed and may include acute or non-acute specialty stays (Table 1). Given the breadth of the VA’s mission, bedded stays include hospital, nursing facility, mental health facility, or domiciliary stays for homeless Veterans. A single-site hospitalization (SSH) is composed of one or more consecutive stays with an acute treating specialty at a single facility.

**Fig. 2:**
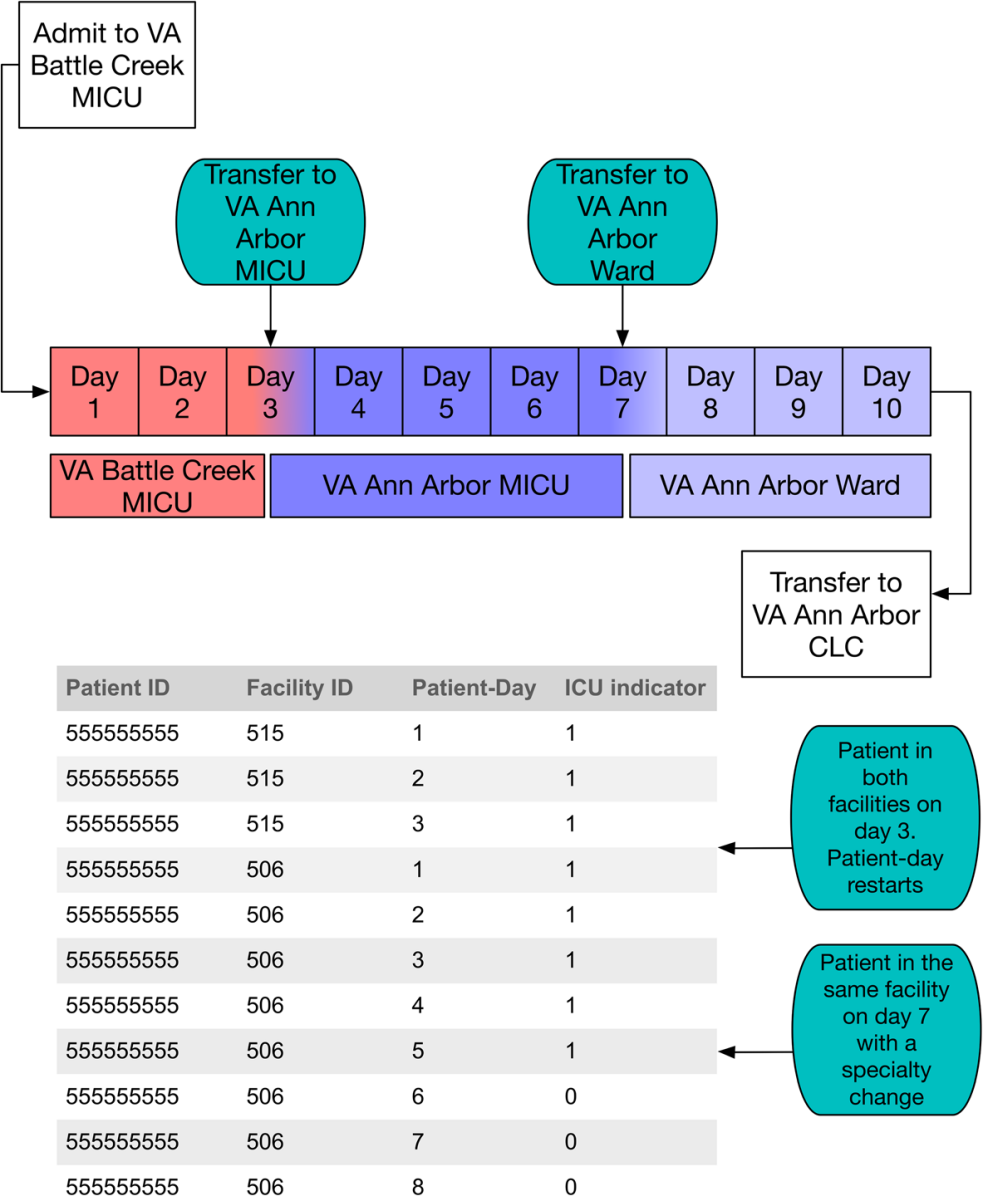
Hypothetical timeline of a patient and data structure. We present a hypothetical patient timeline for a patient with multiple transfers. The patient is admitted to the VA Battle Creek MICU, and transfers to the VA Ann Arbor MICU. Later, they transfer to the VA Ann Arbor ward before ultimately moving to the VA Ann Arbor Community Living Center. In the table, we illustrate how this hypothetical patient timeline would appear in the VAPD

We illustrate the various nesting that exists in the VAPD 2014–2017 using a hypothetical patient timeline and the corresponding data structure in Fig. 2. A patient was admitted to the VA Battle Creek medical intensive care unit (MICU) on day 1. On day 3, the patient was transferred to the VA Ann Arbor MICU for higher level care. The VAPD 2014–2017 would contain two rows for that calendar day, patient-day 3 for Battle Creek and patient-day 1 for Ann Arbor. On day 7, the patient transfers from Ann Arbor MICU to Ann Arbor ward. There would be one row for patient-day 7 because the facility was the same. Additionally, this would be indicated as an ICU day because the patient spent some time in the ICU on that calendar day. Finally, on day 10, the patient transfers to the Ann Arbor Community Living Center (CLC) (a nursing home attached to the VA). The VAPD 2014–2017 would not contain a row for time spent in the nursing home, because it is not acute care.

Thus, the database retains detailed information allowing for analysis at the daily-hospitalization level as well as aggregating by acute SSHs, acute hospitalizations, bedded stays, and overall episodes of care. In the example provided in Fig. 2, the patient had two SSHs (Battle Creek and Ann Arbor), one acute hospitalization (from day 1 to day 10), four bedded stays (Battle Creek MICU, Ann Arbor MICU, Ann Arbor ward, and Ann Arbor CLC) and one overall episode of care. The data can also be rolled up to the patient and hospital facility levels.

#### Abstracting individual laboratory values and vital signs

**Fig. 3:**
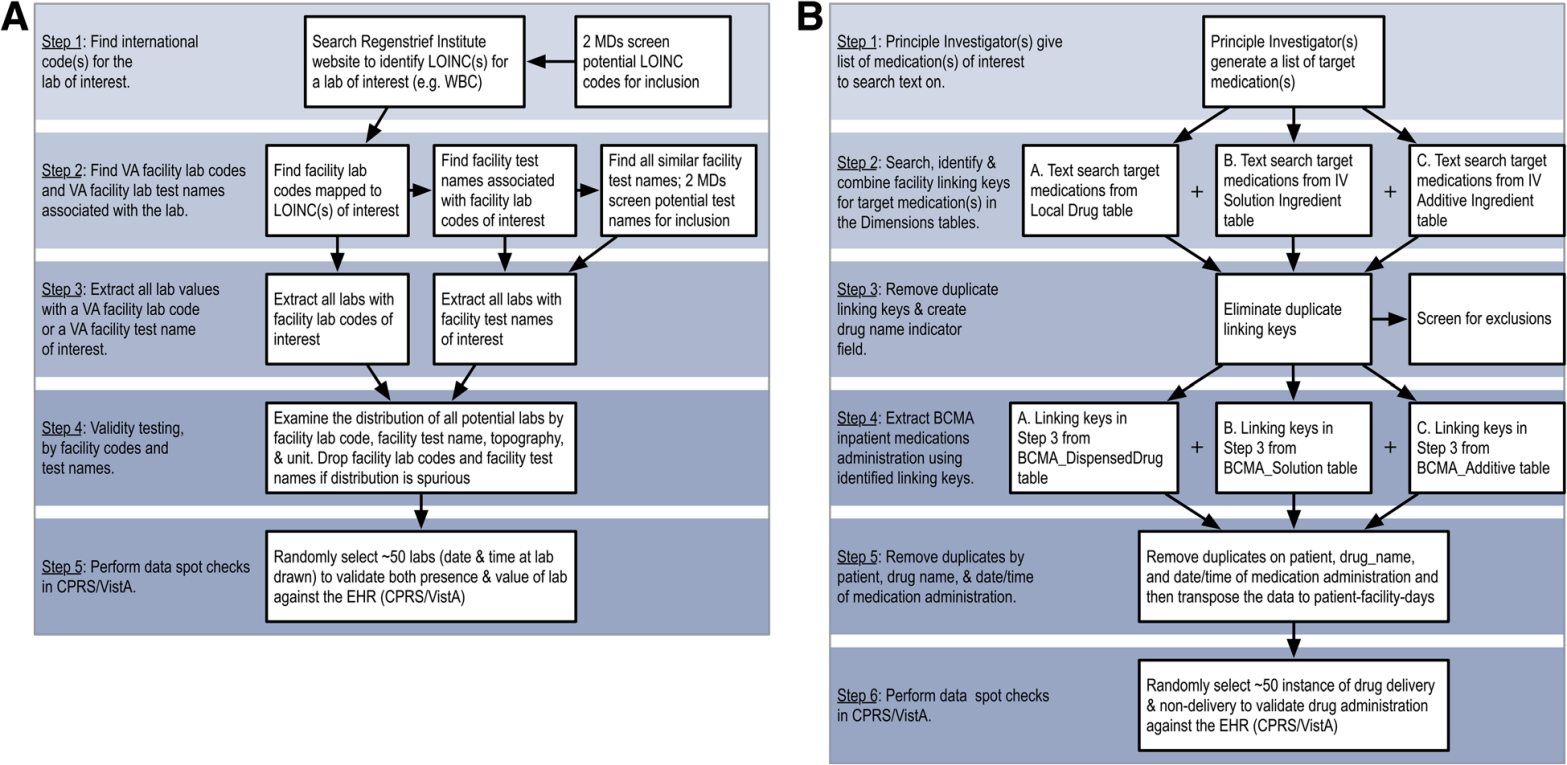
Flow diagram for (**a**) laboratory data extraction from CDW and (**b**) inpatient medications data extraction from CDW. Detailed SOP in (**a**) Appendix B and (**b**) Appendix C of Online Data Supplement

All routine clinical activities including laboratory test values and ward vital signs are documented in VA’s CPRS/VistA. To capture patients’ physiological changes throughout hospitalization, we abstracted high and low values for 16 laboratory tests (e.g. creatinine, albumin) and six vital signs (e.g. temperature, mean arterial pressure), for each patient-facility-day in the VAPD 2014–2017. The complete list of VAPD 2014–2017 laboratory tests and vital signs are reported in Additional file 1: Appendix B of the Online Data Supplement. Because facility laboratory tests are documented differently across individual VA facilities, we have developed and refined a standardized approach to identifying, extracting, and spot checking laboratory values in the VA patient charts. Specifically, we extracted laboratory test values by searching both the Logical Observation Identifiers Names and Codes (LOINC) [12] codes and text fields for facility laboratory test names in CDW (Table 1). Therefore, laboratory tests that might be missed or misclassified by LOINC codes were captured by facility laboratory test names (Fig. 3a). Past work and careful inspection of the data have shown variable penetration of LOINC code – variable over time, by code, and by hospital [10]. Vital signs were extracted from CDW using only the vital types from the Vital Signs table in the CDW Vital Signs domain [10].

After data extraction, we standardized facility laboratory test names, units and comparability. We then aggregated laboratory tests by facility laboratory test names, topography, and units to evaluate distributions for each laboratory test to check value ranges. At least two clinicians reviewed the list of LOINC codes, facility laboratory test names, and result distributions for accuracy. After matching the final laboratory test results and vital signs to the VAPD 2014–2017, at least 50 spot checks were performed by randomly selecting several patient-facility-dates and times on which the laboratory was drawn to validate in the CPRS/VistA systems for data accuracy. For a detailed guide on how we obtained laboratory test values and vital signs, a Standard Operating Procedure (SOP) is included in Additional file 1: Appendix B of the Online Data Supplement and online [5].

#### Abstracting pharmacy data

All VA inpatient medication administrations are recorded using the Bar Code Medication Administration (BCMA) domain and can be extracted from CDW on the patient-facility-day level. The BCMA domain includes local and national drug names, drug class, dosage, route and time of drug administration. For the VAPD 2014–2017, the drugs of interest include antibiotic, vasoactive, sedative, analgesic, paralytic, and other drugs. We first reviewed each drug of interest by VA drug class [13], then extracted data from CDW using text searches for drug names. We conducted text searches for the drugs in any data field that contains drug name information among all medication tables. Drug names were hand-typed in many tables in CDW, and therefore required extensive data cleaning. For example, we assigned any local and national drug names both with and without dosage that contains the word “Rifaximin” to have an indicator of Rifaximin. We excluded drugs containing the words “research” or “study” that also could have showed up in the drug name text searches (Fig. 3b). For a more detailed guide to our approach for extracting inpatient medications from CDW, the SOP is included in Additional file 1: Appendix C of the Online Data Supplement and online [5]. We further grouped antibiotic medications and route of intake into multiple classifications (Additional file 1: Appendix D, Online Data Supplement). At least 50 spot checks were performed by randomly selecting several patient-facility-days to validate in CPRS/VistA systems [7] for data accuracy. The final dataset includes indicators for each drug of interest on the patient-facility-day level, then these indicators were joined to the VAPD 2014–2017.

#### Sepsis definitions

Because sepsis is a research focus for our group, we have included in the VAPD 2014–2017 three indicator variables for sepsis, using three common claims-based or EHR-based methods for identifying sepsis: (1) modified Angus criteria [14, 15] – concurrent diagnostic codes for infection and acute organ dysfunction, or an explicit code for severe sepsis or septic shock; (2) an EHR-based, diagnostic-code-independent definition [16] developed by the Centers for Disease Control and Prevention (CDC) in which the patient has concurrent evidence of infection (blood culture drawn plus systemic antibiotics prescribed) and acute organ dysfunction (e.g. abnormal laboratory values, treatment with vasopressors, treatment with invasive mechanical ventilation). Because U.S. hospitals transitioned from International Classification of Diseases, Ninth Revision, Clinical Modification (ICD-9-CM) codes to International Classification of Diseases, Tenth Revision, Clinical Modification (ICD-10-CM) codes in October 2015, modified Angus criteria had to be converted to ICD-10-CM-based criteria for subsequent hospitalizations. We converted these claims-based sepsis definitions to ICD-10-CM criteria through forward and backward mapping using Center for Medicare and Medicaid Service’s general equivalency mapping [17], and manually reviewed all ICD conversions to ensure their appropriateness in a prior publication [18].

#### Risk score definitions

The VAPD 2014–2017 allows for the calculation of robust illness severity scores. For internal risk-adjustment, VA uses an illness severity measure (the VA illness severity score), which is predicted 30-day mortality based on several variables (age, admission diagnosis category, 30 comorbid conditions, and 11 laboratory values). This severity score performs similarly to APACHE IV, with a C-statistic of 0.874 [19]. Each of the variables used to generate the VA illness severity score are captured in the VAPD 2014–2017. Thus, researchers can generate predicted 30-day mortality, as in Prescott et al. [20]. We elected to provide the components rather than the composite illness scores so that users can generate illness severity scores most appropriate for the research question at hand.

#### Organ dysfunction

The VAPD 2014–2017 includes four (of six) organ based component scores (coagulation, liver, cardiovascular, and renal) of the Sequential Organ Failure Assessment (SOFA). Each organ component is scored from 0 to 4, with higher scores indicating more severe organ dysfunction utilizing the worst physiologic values in one calendar day [21]. We use a modification based on the NICE-SUGAR [22] trial that incorporates a more extensive list of vasopressors for the cardiovascular system; details are in Additional file 1: Appendix A of the Online Data Supplement. We did not attempt to include the central nervous system organ component score given the significant variability in the recording of the Glasgow Coma Score in sedated patients [23]. We have not included the respiratory organ component score because there does not yet appear to be a reliable way to distinguish mechanical ventilation at the day-by-day level; however, mechanical ventilation appears to be reliably detected at the hospitalization level using procedure codes, as are used, for example, in the CDC EHR-based sepsis definition.

All data management and analyses under methodology were conducted using SQL and SAS (SAS Institute), version 9.4. Analyses from the VA were approved by the IRB of the VA Ann Arbor Health System.

## Results

### Hospitalization and patient characteristics

We identified 565,242 SSHs in 2014; 558,060 SSHs in 2015; 553,961 SSHs in 2016; and 550,236 SSHs in 2017 at 141 VA hospitals (Table 2). With the slight decrease in the number of SSHs from 2014 to 2017, the number of unique patients and patient-facility-days also decreased. Majority of SSHs occurred in teaching facilities. The proportion of patient-facility-days in an ICU decreased from 13.8% in 2014 to 13.2% in 2017. The patients were predominantly male (~ 94%) and had a mean age of about 66 years (SD = 13). The median length of stay was four days for all study years.

**Table 2:** Veterans Affairs patient database facility, single-site hospitalization and patient characteristics, 2014–2017

	2014	2015	2016	2017
Facility Characteristics				
Facilities^a^, *N*	141	139	138	141
Teaching, *N* (%)	60 (42.6)	61 (43.9)	61 (44.2)	63 (44.7)
Region				
South	52 (36.9)	52 (37.4)	53 (38.4)	55 (39.0)
West	27 (19.2)	26 (18.7)	25 (18.1)	25 (17.7)
Midwest	37 (26.2)	36 (25.9)	36 (26.1)	36 (25.5)
Northeast	25 (17.7)	25 (18.0)	24 (17.4)	25 (17.7)
Single-site Hospitalizations, *N*				
Small (< 2000)	50 (35.5)	50 (36.0)	47 (34.1)	50 (35.4)
Medium (2000-5000)	39 (27.6)	41 (29.5)	37 (26.8)	39 (27.7)
Large (> 5000)	52 (36.9)	48 (34.5)	54 (39.1)	52 (36.9)
Single-site Hospitalization Characteristics				
Single-site Hospitalizations, *N*	565,242	558,060	553,961	550,236
Length of stay, median (IQR)	4 (2, 6)	4 (2, 6)	4 (2, 6)	4 (2, 6)
In-hospital Mortality, *N* (%)	9754 (1.7)	8913 (1.6)	8255 (1.5)	7936 (1.4)
30-day Mortality, *N* (%)	25,070 (4.4)	23,709 (4.2)	23,093 (4.2)	22,788 (4.1)
30-day Readmission, *N* (%)	85,249 (15.3)	87,209 (15.9)	86,227 (15.8)	84,722 (15.6)
Patients, *N*	373,045	368,837	365,777	364,615
Age, mean (SD)	65.5 (13.3)	65.7 (13.2)	66.0 (13.2)	66.4 (13.2)
Male, *N* (%)	351,298 (94.2)	346,961 (94.1)	343,132 (93.8)	341,882 (93.8)
CDC EHR-based Sepsis Single-site Hospitalization Characteristics				
CDC EHR-based Sepsis Single-site Hospitalizations, *N* (%)^b^	26,882 (4.8)	26,559 (4.8)	27,263 (4.9)	26,808 (4.9)
In-hospital Mortality, *N* (%)	4088 (15.2)	3830 (14.4)	3608 (13.2)	3490 (13.0)
30-day Mortality, *N* (%)	5300 (19.7)	5192 (19.5)	5089 (18.7)	5111 (19.1)
Patient-Facility-Day Characteristics				
Patient-facility-days, *N*	3,170,230	3,086,091	3,021,708	2,961,408
ICU patient-facility-days, *N* (%)	437,627 (13.8)	425,198 (13.8)	401,999 (13.3)	391,454 (13.2)

Abbreviations: ICU, intensive care unit; IQR, interquartile range

30-day mortality was calculated from admission date

30-day readmission rates were calculated from total live discharges

^a^Facility Ns are not consistent across study years because some facilities are newly opened and other older facilities close

^b^Denominator for % is total single-site hospitalizations for each year

### Mortality and readmissions

To illustrate some of the potential applications of the VAPD 2014–2017, we examined in-hospital mortality, 30-day mortality, and 30-day readmission rate after a live discharge. The in-hospital mortality declined from 1.7% in 2014 to 1.4% in 2017; 30-day mortality rate also declined from 4.4% in 2014 to 4.1% in 2017. Whereas 30-day readmission rate increased from 15.3% in 2014 to 15.6% in 2017.

### Organ dysfunction days

Organ failure is defined by having a SOFA score of 3 or 4. The VAPD 2014–2017 consists of 123,378 patient-facility-days of cardiovascular failure; 204,370 patient-facility-days of coagulation failure; 92,716 patient-facility-days of liver failure; and 653,349 patient-facility-days of renal failure.

There were 157,563 patient-facility-days and 107,512 (4.8%) single-site hospitalizations that met the Center for Disease Control and Prevention’s Electronic Health Record-based retrospective definition of sepsis, also known as the Adult Sepsis Event definition [16]. Among hospitalizations meeting EHR sepsis criteria, there were 18,114 patient-facility-days of cardiovascular failure; 9312 patient-facility-days of coagulation failure; 5779 patient-facility-days of liver failure; and 19,644 patient-facility-days of renal failure.

## Discussion

The VAPD 2014–2017 represents a large, standardized collection of granular data from a heterogeneous nationwide healthcare system. Data from 1,052,770 distinct patients; 2,227,499 single-site hospitalizations; 141 facilities and 12,239,437 patient-facility-days are standardized and comparable. These include 355,976 distinct single-site hospitalizations involving critical illness. The longitudinal relationship among rehospitalizations and other care needs can be studied, as can the variation in experience and outcomes between hospitals and larger administrative units.

A major challenge during the construction of the VAPD 2014–2017 was the variability in coding practices across hospitals and over time, despite the nominal presence of a standardized ontology and use of LOINC codes. In the end, EHRs are not used primarily for research or even for system-level profiling. They are used to deliver care to millions of patients, and the exigencies of that care provision at bedside or laboratory often trump the lofty aspirations for standardization of system designers far removed in time, space, and location. Standardized data extraction required iterative interaction between informatics experts and practicing clinicians to validate the information. While some aspects of this procedure could be automated, as noted in the standard operating procedures, we found that expertise from multiple sources (both informatics and clinical) was necessary to achieve reliable data. In VA—as in every health system the authors have been engaged with—work-as-done often proved quite different from work-as-imagined. To support the sorts of science for which the VAPD 2014–2017 is intended, work-as-done needs to be accurately represented.

We have prioritized comparability and openness in several aspects of this work. To make visible this distinction between work-as-imagined and the work-as-done in VA hospitals, we have made the code necessary for data extraction available as a part of this publication. This code may be of interest to individuals working in other hospital systems regardless of direct comparability of their goal or data systems. We, the authors, are committed to collaborating around and sharing these data to maximize their value to improve Veterans and others’ health and health care, to the greatest degree consistent with current Veterans Administration regulations and policy. However, at the time of this writing it is not consistent with policy to release these data into the public domain. To facilitate comparisons, wherever there is overlap, we have also harmonized all measurement units and cut-off points in the VAPD 2014–2017 data to the Adult Patient Database (APD) of the Australian and New Zealand Intensive Care Society’s Centre for Outcome and Resource Evaluation (ANZICS CORE) [24, 25]. By simply adopting an external standard and harmonizing to it—rather than demanding a complex bilateral negotiation—we hope to speed more widespread data comparability efforts to advance scientific research and thereby patient care.

The VAPD 2014–2017 is intentionally designed to open the black-box of within-hospital experience. In particular, many existing databases either contain information only about the hospitalization as a whole, or include physiologic measures only on the first day of the hospitalization. Such data are superb for analyzing risk-adjusted outcomes, as events that occur within the hospital are reasonably assumed to be influenced by the hospital. However, such data are of limited use for evaluating the evolution of patient trajectories within the hospital or the way those trajectories predict subsequent outcomes. Yet a sense of the patients’ in-hospital course is routinely used by clinicians to make ongoing treatment adjustments and decisions, and to prognosticate. We believe the VAPD 2014–2017 may support analyses to help inform such decisions.

The VAPD 2014–2017 has several limitations. Most notably, it represents only one system. While the VA is nationwide, provides inpatient care comparable in quality to other U.S. systems [26], and has longer term outcomes similar to other U.S. systems [27], it is only one system. To reduce this limitation, we have harmonized to the standards of an existing binational database in the hopes of facilitating collaborative cross-system research. The VAPD 2014–2017 draws heavily from data available in structured fields in the VA. While it is naturally extensible to the data in unstructured fields (e.g. modes of mechanical ventilation), such data are not yet available. Finally, the VAPD 2014–2017 requires updating to remain current, both to ensure it reflects current practice and epidemiology, and because inpatient practices will change, requiring constant surveillance of the underlying data extraction codes for relevance, reliability, and completeness.

The VAPD will continue to be built beyond 2017, thus requires routine maintenance and enhancement of its data quality and accuracy. We have built a series of processes to detect changes in the average values or rates of missingness of tests over time at both the system and the individual-hospital level, to prompt investigation to distinguish a true change in practice from changes in the CDW. Our research focuses mainly involve sepsis and critical care, thus a reliable mechanical ventilation is essential in many aspects of our studies. An on-going attempt is to better distinguish mechanical ventilation at the day-by-day level, we plan to explore other methodology in addition to using procedure codes. Our team also plans to tailor the VAPD to study other research questions such as organ failure and medication usage.

## Conclusion

The VAPD 2014–2017 represents a direct resource for studying the evolution of inpatient physiology during both acute and critical illness. It also represents a resource of code and standard operating procedures documenting an approach to achieving reliable, comparable data over years and across a broad system. It is our hope that both its specific instantiation, and the open availability of the procedures used and lessons learned to build that instantiation will be of use.

## Additional file

Additional file 1: **Appendix A.** VAPD definitions for standardized nomenclature and data elements. **Appendix B.** Standard operating procedure for laboratory data extraction. **Appendix C.** Standard operating procedure for medications data extraction. **Appendix D.** Antibiotic drug classifications reference. (PDF 629 kb)

## Abbreviations

AHA: American Hospital Association
ANZICS CORE: Australian and New Zealand Intensive Care Society’s Centre for show Outcome and Resource Evaluation
APACHE: Acute Physiology and Chronic Health Evaluation
APD: Adult Patient Database
BCMA: Bar Code Medication Administration
CCS: Clinical Classifications Software
CDC: Center for Disease Control and Prevention
CDW: Corporate Data Warehouse
CLC: Community Living Center
CPRS: Computerized Patient Record System
EHR: Electronic Health Records
ICD-9-CM: International Classification of Diseases, Ninth Revision, Clinical Modification
ICD-10-CM: International Classification of Diseases, Tenth Revision, Clinical Modification
ICU: Intensive Care Unit
LOINC: Logical Observation Identifiers Names and Codes
MICU: Medical Intensive Care Unit
MIMIC: Medical Information Mart for Intensive Care
PHC4: Pennsylvania Health Care Cost Containment Council
SOFA: Sequential Organ Failure Assessment
SOP: Standard Operating Procedure
SSH: Single-site Hospitalization
VA: Veterans Affairs
VAPD 2014–2017: Veterans Affairs Patient Database 2014–2017
VISTA: Veterans Information Systems Technology Architecture
