# Supplementary material for: Veterans Affairs patient database (VAPD 2014–2017): building nationwide granular data for clinical discovery
Source: BMC Med Res Methodol. 2019 May 8;19:94. doi: 10.1186/s12874-019-0740-x (PMC6505066; doi:10.1186/s12874-019-0740-x)
Supplement: Supplementary file 1 — Appendix A. VAPD definitions for standardized nomenclature and data elements. Appendix B. Standard operating procedure for laboratory data extraction. Appendix C. Standard operating procedure for medications data extraction. Appendix D. Antibiotic drug classifications reference. (PDF 629 kb) [file 12874_2019_740_MOESM1_ESM.pdf]

ONLINE DATA SUPPLEMENT

Title: Veterans Affairs patient database (VAPD): building nationwide granular data for clinical discovery

Authors: Xiao Qing Wang, MPH, Brenda M. Vincent, MS, Wyndy Wiitala, PhD, Kaitlyn A. Luginbill, MPH, Elizabeth M. Viglianti, MD, MPH, Hallie C. Prescott, MD, MSc, Theodore J. Iwashyna, MD, PhD

Table of Contents

Appendix A: VAPD definitions for standardized nomenclature and data elements

Appendix B: Standard operating procedure for laboratory data extraction

Appendix C: Standard operating procedure for medications data extraction

Appendix D: Antibiotic drug classifications reference

Appendix A: VAPD definitions for standardized nomenclature and data elements

VAPD Standardized Nomenclature

**Term**	**Conceptual definition**
VAPD	Veterans Affairs Patient Database (how we refer to the database throughout the paper)
Patient-facility-day	An individual day that a patient spent in the hospital, defined as calendar date. A patient-facility-day may be associated with multiple hospitalizations if a patient is transferred between hospitals
Patient	The term used to indicate the individual person (as opposed to participant, subject, Veteran, etc.)
Hospital	The physical place (site or facility) where the patient was treated
SSH	Single-site Hospitalization
ICU	Intensive Care Unit
Laboratory	The physical lab (site) where the testing machines exist
Laboratory test	The concept of the test conducted by a laboratory (e.g. albumin, bilirubin). There may be various names and clinical synonyms for an individual lab test
Facility laboratory test name	The name used to identify a lab test at a specific site (e.g. white blood cell count, WBC)
Test results	The result of a laboratory test
Laboratory test synonyms	Other clinical names for the same laboratory test (e.g. blood gas, carbon dioxide both map to the same lab test)
Facility laboratory code	Facility-specific code linked to lab test names (the variable name used in the Corporate Data Warehouse is LabChemTestSID)
Facility LOINC	Facility-specific code linked to LOINC codes (the variable name used in the Corporate Data Warehouse is LOINCSID)
Bedded stay	Any stay in a healthcare facility where a patient is provided a bed, including hospital, nursing facility, mental health facility, or domiciliary for homeless Veterans
Specialty stay	A portion of a bedded stay defined by the treating specialty. Each bedded stay is composed of one or more specialty stays
Acute specialty stay	A specialty stay that is for an acute medical condition
Non-acute specialty stay	A specialty stay that is not for an acute medical condition
Hospitalization	One or more consecutive acute specialty stays
Specialty transfer	Occurs when a patient’s care is transitioned from one treating specialty to another treating specialty
Topography	A specific description of an anatomic region of the body where lab specimen was drawn (e.g. arterial blood, plasma, blood, serum)

Data Elements

Clinical Data Type	Concepts	Variable Level
Demographics	Age, race, sex	Patient
Laboratory tests	Daily high/low values for tests ordered: Albumin, bilirubin, creatinine, platelets, potassium, white blood cell count, urea, bicarbonate, sodium, lactate, glucose, hematocrit, hemoglobin, pH, PaCO2, & PaO2	Patient-facility-day
Vital signs	Respiratory rate, mean arterial blood pressure, heart rate, systolic blood pressure, diastolic blood pressure, core temperature	Patient-facility-day
Hospital admission	Admission and discharge dates, length of stay	Hospitalization
Severity scores	30 Elixhauser comorbidities, Inpatient Evaluation Center (IPEC) severity score	Hospitalization
ICU admission	ICU indicator, ICU length of stay, ICU admission and discharge dates	Hospitalization, patient-facility-day
Discharge disposition	Discharge to home, transfer to another acute care, death	Hospitalization
Death status	Death date as of the end of the calendar year and an indicator for death in the hospital	Patient, hospitalization
Hospital characteristics	State, region, number of operating beds, number of beds, indicator for teaching hospital, facility level,	Hospital
Diagnoses	ICD-9 for primary diagnosis and up to 14 additional diagnoses, the single-level Clinical Classification Software category for primary diagnosis	Hospitalization
Sepsis definitions	Angus definition of infection, Angus definition of acute organ dysfunction, Angus definition of explicit diagnosis, Angus definition of sepsis, CDC EHR-based sepsis definition	Hospitalization
Antibiotics	Antibiotic name and route (i.e., Penicillin_IV, Amoxicillin_PO) were grouped into multiple antibiotic classes which includes Penicillin, 1st – 4th Generation Cephalosporin, Fluoroquinolone, Antiviral, Antifungal, etc. A complete list and classification of Antibiotics is provided in Appendix D.	Patient-facility-day
Microbiology	Blood culture, other micro labs (e.g., urine, sputum)	Patient-facility-day
Vasoactive drugs	Norepinephrine, epinephrine, phenylephrine, dopamine, vasopressin	Patient-facility-day
Sedative drugs	Propofol, Ketamine, Midazolam, Lorazepam	Patient-facility-day
Paralytic drugs	Cisatracurium, Vecuronium, Etomidate	Patient-facility-day
Analgesic drugs	Fentanyl, Morphine, Hydromorphone (Dilaudid)	Patient-facility-day
Other drugs	Lactulose, Rifaximin	Patient-facility-day
Prior hospitalization history	Number of hospitalizations for the patient in the prior calendar year	Hospitalization
Readmissions	Date of readmitting hospitalizations in 30 and/or 90 days, diagnosis type of readmitting hospitalizations	Hospitalization

Appendix B: Standard operating procedure for laboratory data extraction

Goal: Identify labs drawn for a patient during an inpatient stay on a day-by-day basis. This SOP provides step-by-step instructions on how to extract pharmacy data from the Corporate Data Warehouse (CDW). Medications of interest are extracted annually by calendar year.

Data Organization:

- CDW: Data stored in the Corporate Data Warehouse are organized as relational tables. Data are separated into multiple domains (such as vital signs, laboratory, inpatient, outpatient, etc.) and tables within each domain. Linking keys (ending in 'SID') are used to reassemble data elements of interest to create tables for analysis.
- Dimension (Dim) tables: Supporting tables which hold meta data. For example, the inpatient diagnosis tables would contain a key for a diagnosis code and the diagnosis dim table would provide the actual diagnosis code value.
- LabChem Domain: Domain containing laboratory tests and results.
- Field: A column of data in a table.
- PatientICN: Unique patient ID. Each facility has an ID for a patient (PatientSID) so that a patient seen at multiple facilities would have multiple PatientSIDs but the PatientICN is unique at the patient-level.

<u>LOINC Codes</u>

Regenstrief LOINC Database: https://loinc.org/

- LOINC codes are a universal code system for tests, measurements, & observations
- Format: a formal, distinct, and unique 6-part name given to each term for tests/observation identity (e.g. LOINC=66768-3).
- Analyst must create an account first before searching the database, https://search.loinc.org/searchLOINC/
- Example: searching "White Blood Cell" will give the following LOINC codes: 66768-3, 12227-5, 61182-2, etc.

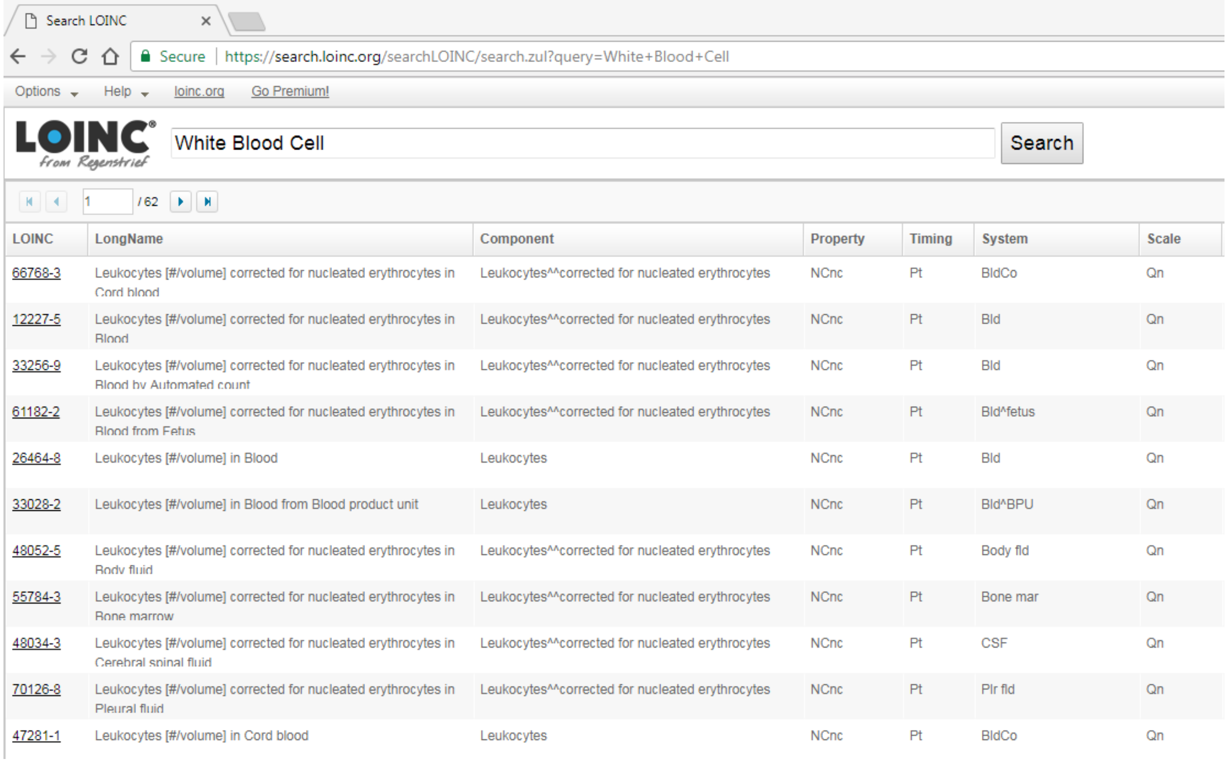

LOINC	LongName	Component	Property	Timing	System	Scale
66768-3	Leukocytes [#/volume] corrected for nucleated erythrocytes in Cord blood	Leukocytes^^corrected for nucleated erythrocytes	NCnc	Pt	BldCo	Qn
12227-5	Leukocytes [#/volume] corrected for nucleated erythrocytes in Blood	Leukocytes^^corrected for nucleated erythrocytes	NCnc	Pt	Bld	Qn
33256-9	Leukocytes [#/volume] corrected for nucleated erythrocytes in Blood by Automated count	Leukocytes^^corrected for nucleated erythrocytes	NCnc	Pt	Bld	Qn
61182-2	Leukocytes [#/volume] corrected for nucleated erythrocytes in Blood from Fetus	Leukocytes^^corrected for nucleated erythrocytes	NCnc	Pt	Bld^fetus	Qn
26464-8	Leukocytes [#/volume] in Blood	Leukocytes	NCnc	Pt	Bld	Qn
33028-2	Leukocytes [#/volume] in Blood from Blood product unit	Leukocytes	NCnc	Pt	Bld^BPU	Qn
48052-5	Leukocytes [#/volume] corrected for nucleated erythrocytes in Body fluid	Leukocytes^^corrected for nucleated erythrocytes	NCnc	Pt	Body fld	Qn
55784-3	Leukocytes [#/volume] corrected for nucleated erythrocytes in Bone marrow	Leukocytes^^corrected for nucleated erythrocytes	NCnc	Pt	Bone mar	Qn
48034-3	Leukocytes [#/volume] corrected for nucleated erythrocytes in Cerebral spinal fluid	Leukocytes^^corrected for nucleated erythrocytes	NCnc	Pt	CSF	Qn
70126-8	Leukocytes [#/volume] corrected for nucleated erythrocytes in Pleural fluid	Leukocytes^^corrected for nucleated erythrocytes	NCnc	Pt	Plr fld	Qn
47281-1	Leukocytes [#/volume] in Cord blood	Leukocytes	NCnc	Pt	BldCo	Qn

CDW LOINC & LabChemTest Tables

There are two CDW tables to look up potential labs that are used, to capture as many lab observations as possible:

1. Dim.LOINC: LOINC & LOINCSID
    - Each LOINC can have multiple LOINCSIDs, because each Sta3n (Facility_ID) has a unique LOINCSID for a LOINC code.
    - Find all LOINCSIDs associated with the given LOINC code.
2. Dim.LabChemTest: LabChemTestName and LabChemTestSID
    - Each LabChemTestName can have multiple LabChemTestSIDs depending on Sta3n.
    - Find all LabChemTestSIDs associated with the given lab name.

Dim.LOINC

Results | Messages

	LOINCSID	Sta3n	LOINCIEN	LOINC	Component
1	800357403	678	26464	26464-8	LEUKOCYTES
2	800306119	664	26464	26464-8	LEUKOCYTES
3	800280289	649	26464	26464-8	LEUKOCYTES
4	800385828	691	26464	26464-8	LEUKOCYTES
5	800243669	644	26464	26464-8	LEUKOCYTES
6	800166165	600	26464	26464-8	LEUKOCYTES
7	800119712	593	26464	26464-8	LEUKOCYTES
8	800090788	519	26464	26464-8	LEUKOCYTES
9	800020476	501	26464	26464-8	LEUKOCYTES
10	800606394	531	26464	26464-8	LEUKOCYTES
11	800523187	442	26464	26464-8	LEUKOCYTES
12	800212414	605	26464	26464-8	LEUKOCYTES
13	800055280	504	26464	26464-8	LEUKOCYTES
[illegible]	[illegible]	[illegible]	[illegible]	[illegible]	[illegible]

Dim.LabChemTest

Results | Messages

	LabChemTestSID	LabChemTestIEN	Sta3n	LabChemTestName	LabChemPrintTestName
15	1000110365	7794	695	WHITE BLOOD CELL COUNT (DYNACARE)	WBC COUNT (DYNA...
16	1200027979	1480	652	WHITE BLOOD CELL CT	WBC
17	1400012605	151	405	WHITE BLOOD CELL/URINE	WBC/HPF
18	800005098	1	459	WHITE BLOOD CELLS	WBC
19	800222344	6000	575	WHITE BLOOD CELLS	WHITE
20	1400581067	5783	595	WHITE BLOOD CELLS	WBC's
21	1000108537	9845	636	WHITE BLOOD CELLS (Million/mL)(UNIV.)*IC	WBC/mL
22	800237765	10811	691	WHITE BLOOD CELLS (STOOL)	WBC-STL
23	1000097335	6927	671	WHITE BLOOD CELLS (VCB)(182352)	WBC SPUTUM
24	800016430	6536	501	White Blood Cells (WBC)	wbc
25	1000100494	1169	740	WHITE BLOOD CELLS (WBC) STOOL, (008656)	FEC LEU
26	1200072589	1977	516	WHITE BLOOD CELLS Thru 2/12/07	WBC
27	1200079886	1976	516	White Blood Cells*	WBC*

Defining & Extracting New Labs

Step 1: Search Regenstrief Institute Website to Identify LOINC Codes

- Get a list of synonyms and names for a given lab from Principle Investigators (PIs) who have clinical knowledge.
    - Example: Arterial Blood Gas or Venous Blood Gas Labs may have any of the following names:
        - PaCO2, PaO2, PCO2, PO2, blood gas, oxygen, partial pressure, carbon dioxide
- Search the synonyms & names on the LOINC database, then copy and paste the results to an Excel spreadsheet (one lab synonym/name per sheet by their search keyword).
    - Data cleaning: When using multiple synonyms, be sure to de-duplicate across sheets to reduce hand-arbitration burden. For example, the same LOINC codes might show up in PaCO2 and blood gas, so keep only the code for PaCO2 and drop the one for blood gas.
- Identify a list of synonyms and names for a given lab from PIs to exclude:
    - Ask PIs about topography: For example, exclude any LOINC component that contains “cord blood”, “mixed venous”, “capillary”, “airway circuit”. By excluding the incorrect topography, labs will decrease time/burden for PIs.
- Send excel file of LOINC codes and long names for each LOINC code to at least two PIs to individually review which LOINC codes to keep and exclude. The PIs will then return the excel spreadsheet to the analyst with a list of LOINC codes to retain/exclude.
- Analyst makes a list of LOINC codes where PIs disagree and sends to PIs again for consensus.

Step 2: Identifying Facility Lab Codes (LOINCSIDs) & Lab Test Names (LabChemTestSIDs)

LOINCSIDs

- Analyst creates a csv file with all the verified LOINC codes for the specific lab from Step 1
    - Be sure to name by date and add the “as of date” column in which consensus occurred between the two PIs for record keeping purposes

	A	B	C	D	E	F	G	H	I	J	K	L	M	N	O	P	Q	R	S	T	U	V	W
1	LOINC	Jack	Liz	consensus	LongName	Comp	Pr	Timi	Syst	Scale	Met	exUC	exUr	Lform	Rank	SIRank	Class	ShortNa	Type	Order(	DocSec	Copy	as of date
2	11556-8	1	1	1	Oxygen [Partial pressure] in Blood	Oxyg	PP	Pt	Bld	Qn		mm[	mm Hg		87	87	CHEM	pO2 Bld	Lab	Both			10/13/2017
3	11557-6	1	1	1	Carbon dioxide [Partial pressure] in Blood	Carb	PP	Pt	Bld	Qn		mm[	mm Hg		86	86	CHEM	pCO2 Bl	Lab	Both			10/13/2017
4	18767-4	1	1	1	Blood gas studies (set)	Blooc	Cn	-	^Pat	Set					.	.	ATTACH.LAB		Attachment				10/13/2017
5	19214-6	1	1	1	Oxygen [Partial pressure] saturation adjusted to 0.5 i	Oxyg	PP	Pt	BldA	Qn		mm[	mm Hg		.	.	CHEM	pO2 sat	Lab	Observation			10/13/2017
6	19216-1	1	1	1	Oxygen [Partial pressure] saturation adjusted to 0.5 i	Oxyg	PP	Pt	BldV	Qn		mm[	mm Hg		.	.	CHEM	pO2 sat	Lab	Observation			10/13/2017
7	19218-7	1	1	1	Oxygen content in Arterial blood	Oxyg	SC	Pt	BldA	Qn		mol/	mol/L		.	.	CHEM	O2 Ct Bl	Lab	Observation			10/13/2017
8	19220-3	1	1	1	Oxygen content in Venous blood	Oxyg	SC	Pt	BldV	Qn		mol/	mol/L		.	.	CHEM	O2 Ct Bl	Lab	Observation			10/13/2017
9	19254-2	1	1	1	Oxygen [Partial pressure] adjusted to patient's actual	Oxyg	PP	Pt	Bld	Qn		mm[	mm Hg		619	619	CHEM	pO2 ten	Lab	Observation			10/13/2017
10	19255-9	1	1	1	Oxygen [Partial pressure] adjusted to patient's actual	Oxyg	PP	Pt	BldA	Qn		mm[	mm Hg		.	.	CHEM	pO2 ten	Lab	Observation			10/13/2017
11	19258-3	1	1	1	Oxygen [Partial pressure] adjusted to patient's actual	Oxyg	PP	Pt	BldV	Qn		mm[	mm Hg		.	.	CHEM	pO2 ten	Lab	Observation			10/13/2017

    - Additionally, also create a list of LOINC codes that were excluded by PIs. Moving forward for a specific lab pull during a new year, when starting the search.loinc in step 1 again, exclude those LOINC codes that were excluded before to shorten the PIs’ review process.
- Pull these verified LOINC codes from CDW table: dim.loinc → get all the LOINCSIDs for that given lab to be used later, and then save the output as a dflt table. (See example SQL code below)

Example SQL code:

```
select LOINC, Component, Sta3n, LOINCSID
into #loinc
from [CDWWork].[Dim].[loinc]
where loinc in ('76625-3','16324-6','1742-6','1744-2','1743-4','77144-4','44785-4','48134-1','76625-3','77144-4')
--1040 rows

select * from  #loinc
```

LabChemTestSIDs

Next, we need to identify LabChemTestSIDs in CDW. We are not able to rely only on LOINCSIDs because some data tables that we need are linked to LabChemTestSIDs rather than LOINCSIDs. Therefore, once a list of LOINCSIDs is obtained, in order to see what LabChemTestNames are used for the lab of interest, we need to pull in ALL the labs within the study period using those LOINCSIDs. We will later use the PI-verified LabChemTestNames in order to pull the correct labs of interest for patients. LabChemTestNames may be entered inconsistently into CPRS, so it is important to review the list of all LabChemTestNames to ensure that we are pulling the correct labs.

- Pull in all labs using LOINCSID, obtaining the LabChemTestNames field from Dim.labchemtest

```
SELECT a.LabChemTestSID, a.PatientSID, a.sta3n, a.LabChemSpecimenDateTime, a.LOINCSID,  c.labchemtestname
into #labchemtests
FROM  [ORD_[redacted]D].[Src].[Chem_PatientLabChem] AS A
INNER JOIN #loinc b on  a.Loincsid=b.Loincsid
LEFT JOIN [CDWWork].[dim].labchemtest  as c on a.Labchemtestsid=c.Labchemtestsid
WHERE  (a.LabChemSpecimenDateTime >= '20140101'  AND  a.LabChemSpecimenDateTime < '20180101') /*can change dates*/
       and (a.LabChemResultNumericValue is NOT NULL)
--( row(s) affected)

select top (1000) * from #labchemtests

--save above table into dflt table if everything looks ok

select  *
into dflt.ATLlabname_20190404_SW /*change name when saving this table*/
from #labchemtests
--(16750027 row(s) affected)

alter table dflt.ATLlabname_20190404_SW /*copy and paste table name here: dflt.TableName or temp.tablename*/
rebuild partition=ALL
with
(data_compression=page)

/*export above take into SAS. Make sure to remove duplicates by patientsid, sta3n, LabChemSpecimenDateTime, LOINCSID & labchemtestname
then look at frequency. Anything below N=100 can just delete*/
```

- Export dflt.labname_date table into SAS dataset for further management
    - Make sure to remove duplicate patient labs
    - Then get a frequency list of LabChemTestNames
        - In general, for labs with thousands of LabChemTestNames, LabChemTestNames with less than 100 occurrences can be deleted. However, this will vary by lab. So confirm with PI regarding the frequencies prior to deleting.
- Copy and paste frequency table to excel -OR- export the table to either CSV or Excel file using SAS code (below)
- Sort the frequency table by LabChemTestNames from greatest to least frequency.
- Download the file onto a VA PC from VINCI using the VINCI download tool in Applications (https://vaww.vinci.med.va.gov/WebApps/VCFileTransfer/Download)
- Send list of LabChemTestNames frequency to PIs for review
    - Analyst gives frequency list to at least two PIs to review and decide which LabChemTestNames to keep or exclude.
    - After PIs return their individual lists, analyst makes a list of LabChemTestNames where PIs disagree.
    - Analyst then sends updated list back to PIs again for consensus.
    - Once consensus is found, analyst then creates an excel file of all the LabChemTestNames associated with a given lab exactly as they appear in the CDW Dim.LabChemTest table.
    - Note: In the excel file, make sure to first change LabChemTestNames such as '%NEUTROPHILS (CZ)(Dc'd)' to '%NEUTROPHILS (CZ)(Dc''d)' before saving it as csv file.

LabChemTestName
% LYMPHOCYTES
**%NEUTROPHILS (CZ)(Dc"d)**
(IDM) %MONOCYTES
.LEUKOCYTES
.LYMPHOCYTES
.LYMPHOCYTES%
.Lymphocytes,Absolute(SL)
.MONOCYTES
.MONOCYTES (ABSOLUTE)
**.MONOCYTES (PI) DC"ED 082114**
.NEUTROPHILS
.NEUTROPHILS (ABSOLUTE)

  - Additionally, also create a list of LabChemTestNames that were excluded by PIs. Moving forward for a specific lab pull during a new year, when we repeat this step again, we will exclude those LabChemTestNames that were excluded before to shorten the PIs' review process.

- Pull the new LabChemTestNames from CDW table: Dim.LabChemTest → get all the LabChemTestSIDs for that given lab to be used later.

Example SAS code:

```
/*import ABG LabChemTestNames that PIs decided to keep*/
proc import datafile="[redacted]\ABGs\ABG_LabName_10242017.csv"
out=ABG_LabName dbms=csv replace;
getnames=yes;
guessingrows=1234567;
run;

/*pull all LabChemTestSID associated with &abglabname10242017*/
proc sql;
create table temp.abglabchemsid_&todaysDate as
select distinct Labchemtestsid, LabChemTestName, Sta3n
from dim.labchemtest
where labchemtestname in (SELECT LabChemTestName  FROM ABG_LabName);
quit;

/*export to csv */
%ds2csv (
  data=temp.abglabchemsid_&todaysDate,
  runmode=b,
  csvfile="[redacted]s\ABG_LabChemTestSID_&todaysDate.csv"
 );

/*make a macro WBC LabChemSIDs list*/
proc sql;
        select  catt("'", LabChemTestSID, "'")
        INTO :ABGLabChemTestSID10242017 separated by ','
        from temp.abglabchemsid_&todaysDate;
quit;
%put &ABGLabChemTestSID10242017;
```

Step 3: Extract Verified LOINCSIDs & LabChemTestSIDs

- Pull verified LOINCSIDs and LabChemTestSIDs from CDW, exclude those with missing result values.
- Remove duplicate labs.
- Get PatientICN & reformat LabChemSpecimenDateTime to MM/DD/YYYY

Example SQL code:

```
/*pull ABG loincsids and labchemtestsids from CDW for 2014-2017*/
PROC SQL ;
CREATE TABLE abg_2014_2017 (compress = yes) AS
SELECT a.Sta3n,  a.LabChemTestSID, a.PatientSID, a.LabChemSpecimenDateTime, a.LabChemResultNumericValue,
a.TopographySID, a.LOINCSID, a.Units, a.RefHigh, a.RefLow, d.Topography
FROM  [INSERT STUDY NAME].[src].[Chem_PatientLabChem] AS A
INNER JOIN  ABG_loincsid b on  a.Loincsid=b.Loincsid
LEFT JOIN [CDWWork].[Dim].[topography] AS d ON A.TopographySID =D.TopographySID
            WHERE a.LabChemSpecimenDateTime >= &startdate. and a.LabChemSpecimenDateTime < &enddate.

UNION

SELECT a.LabChemSID, a.LabSubjectSID,  a.Sta3n, a.LabPanelIEN, a.LabPanelSID, a.LongAccessionNumberUID,
a.ShortAccessionNumber,
      a.LabChemTestSID, a.PatientSID, a.LabChemSpecimenDateTime, a.LabChemSpecimenDateSID, a.LabChemCompleteDateTime,
a.LabChemCompleteDateSID,
      a.LabChemResultValue, a.LabChemResultNumericValue, a.TopographySID, a.LOINCSID, a.Units, a.RefHigh, a.RefLow,
d.Topography
FROM [INSERT STUDY NAME].[src].[Chem_PatientLabChem] a
INNER JOIN ABG_labchemsid b ON a.labchemtestsid=b.labchemtestsid
LEFT JOIN [CDWWork].[Dim].[topography] AS d ON A.TopographySID =D.TopographySID
    WHERE loincsid=-1 and
     a.LabChemSpecimenDateTime >= &startdate. and a.LabChemSpecimenDateTime < &enddate.

QUIT;

/*remove duplicate labs */
PROC SORT DATA=abg_2014_2017 nodupkey out=abg_mechvent_2014_2017;
BY  patientSID  sta3n  LabChemResultNumericValue LabChemSpecimenDateTime;
RUN;

/*get unique patienticn*/
proc sql;
create table abg_mechvent_2014_2017_V2 (compress=yes) as
select a.*, b.patienticn
from abg_mechvent_2014_2017 a
left join [INSERT STUDY NAME].[src].[CohortCrosswalk] b on a.PatientSID=b.PatientSID ;
QUIT;

/*change patienticn into numeric*/
DATA abg_mechvent_2014_2017_V3  (rename=patienticn2=patienticn compress=yes);
SET abg_mechvent_2014_2017_V2;
patienticn2 = input(patienticn, 10.);
drop patienticn;
LabSpecimenDate=datepart(LabChemSpecimenDateTime); /*convert datetime to date*/
format LabSpecimenDate mmddyy10.;
RUN;
```

Step 4: Look at Frequencies and Descriptive of All Labs

- Examine all lab names, units, topography, and lab result values.
    - Check the percent of missing values for LabChemResultNumericValue.
        - Some missing values for LabChemResultNumericValue are due to the string variable LabChemResultValue, which may contain character values such as ‘>’ and ‘<’ that were coded as missing in the numeric variable (LabChemResultNumericValue). Confirm with PIs the correct way to recode the missing values for LabChemResultNumericValue. Additionally, create a new flag variable (LabChemResultNumericValue_flag) to indicate the values that were recoded.
    - Standardize the unit formats (create clean_unit variable) and exclude incorrect ones
        - Make units all capital letters, delete ‘.’ (periods), compress any spaces
        - PI will decide whether to exclude those with unclear or wrong units

- Look at topography and exclude incorrect ones
  - Note that we have compared DefaultTopography and CollectionSample from Dim.CollectionSample table and found that Topography from Dim.Topography has the most complete non-missing information
  - PIs decide which to keep or exclude
- Keep only those with frequencies > 100 (but confirm with PI first if that's still the case before deleting)
- Separate dataset into those with units vs. those without units, and perform the following on both datasets:
  - Aggregate by LabChemTestName, Topography, and clean_unit:
  - Clean up LabChemTestNames; for example:

%let LYMPHOCYTE_AUTO =('LYMPH (AUTO)','LYMPH (AUTO)','LYMPHS (AUTO)', 'LYMPHOCYTE (AUTO)');
%let LYMPHOCYTE_MANUAL=('LYMPHS (MANUAL)','LYMPHS (MAN)','LYMPHSMANUAL','LYMPHOCYTE MANUAL');

- Investigate normal ranges for lab values: RefHigh and RefLow. Some LabChemTestSIDs have multiple RefHigh and RefLow values. Keep them separate, do not aggregate or deduplicate.
- Calculate the median, 10th percentile, 90th percentile of RefHigh and RefLow for each aggregated LabChemTestName, Topography, and clean_unit
- Sort by LabChemTestName, clean_unit, RefLow, RefHigh
- Send to two PIs to individually review
- Analyst creates list where PIs disagree, and then sends to PIs again to confirm. PIs are trying to ascertain if the specific lab seems plausible for the lab of interest and will flag those that appear clinically unlikely.

	A	B	C	D	E	F	G	H	I	J	K
1	Topography	Labchemtestname	clean_unit	Median	P10	P90	RefLow	RefHigh	total_num_agglabchemtest	include	weird
2	ARTERIAL BLOOD	ABGCTO2	PERCENT	14.9	11	18.7	16	21.5	273	0	
3	ARTERIAL BLOOD	ABGCTO2	PERCENT	14.4	11	18.7	17.5	23	8099	0	
4	ARTERIAL BLOOD	ABGFCOHB	PERCENT	1.3	0.8	2.2	.	.	8366	0	
5	ARTERIAL BLOOD	ABGFIO2	PERCENT	40	21	80	.	.	8271	0	
6	ARTERIAL BLOOD	ABGFO2HB	PERCENT	94.1	86.3	96.5	90	95	639	1	
7	ARTERIAL BLOOD	ABGPCO2(T)	MM/HG	42.1	32.3	59.4	35	45	8377	1	
8	ARTERIAL BLOOD	ABGPO2(A/AT)E	MM/HG	71.8	27.8	241.3	.	.	539	1	
9	ARTERIAL BLOOD	ABGPO2(A/AT)E	PERCENT	50.55	23.5	74.5	.	.	542	0	
10	ARTERIAL BLOOD	ABGPO2(T)	MM/HG	88.9	61.8	210	80	100	8363	1	
11	ARTERIAL BLOOD	ABGSO2	PERCENT	97.3	91.7	99.6	94	100	8377	1	
12	ARTERIAL BLOOD	ANCILLARY PCO2	MM/HG	39	30.2	49.1	32	46	5451	1	
19	BLOOD	AT PCO2(T)	MM/HG	43.7	33	58.5	35	45	1171	1	
20	ARTERIAL BLOOD	AT PO2	MM/HG	105	65	344	80	105	6825	1	
21	BLOOD	AT PO2	MM/HG	38	27	120	80	105	1633	1	1
22	ARTERIAL BLOOD	AT PO2(T)	MM/HG	96	64	227	80	105	5439	1	
23	BLOOD	AT PO2(T)	MM/HG	34	26	57	80	105	1041	1	1
24	ARTERIAL BLOOD	AT TCO2 (MEASURED)	MMOL/L	24	22	28	23	27	562	0	
25	ARTERIAL BLOOD	AT TCO2 (MEASURED)	MMOL/L	26	22	30	24	29	744	0	
26	BLOOD	AT TCO2 (MEASURED)	MMOL/L	26	22	31	24	29	1747	0	
27	ARTERIAL BLOOD	AT TCO2 CALCU	MMOL/L	25	21	32	19	24	2976	0	
28	BLOOD	AT TCO2 CALCU	MMOL/L	26	22	31	19	24	819	0	
29	ARTERIAL BLOOD	AT TCO2 CALCU	MMOL/L	25	21	31	23	27	4362	0	

- Make histograms of those that appear unlikely, so that PIs can re-evaluate if they are plausible for the lab of interest. It depends on a particular test, but some need to be displayed with a bin width of 10.
- For those without units, look at the descriptive statistic and let the PIs decide whether to drop or keep them in.

Example SAS code:

```
/*clean up and standardize units*/
DATA  pco_2014_C2; /*2,655,639*/
SET   pco_2014_C;
Units2=upcase(units); /*turn all units into uppercase*/
units3=compress(Units2,'.'); /*removes '.' in units*/
clean_unit=compress(units3); /*removes all blanks (by default - specify options to remove other chars)*/
drop units2 units3;
RUN;

/*Look at Unit and Topography frequencies and PIs help decide which to exclude or keep*/
/*clean/drop units & topography*/
DATA  pco_2014_C3;
SET  pco_2014_C2;
if clean_unit in ('FAHRENHEIT','L/MIN','LPM','CC/100ML','C','OBS','%CAL','326','DEGREESC','G/DL','MG/DL') then delete;
if topography in ('MIXED VENOUS','URINE','MIXED VENOUS BLOOD','VENOUS BLOOD (MIXED)','PLEURAL FLUID','MIXED
VEN/ART BLD','SWAN-GANZ CATHETER','BILE','FECES','PERITONEAL FLUID') then delete;
if clean_unit='VOL%' or clean_unit='%' or clean_unit='%MEASURED' then clean_unit='PERCENT';
if topography='SERUM-UNK' or topography='serum' then topography='SERUM';
RUN;

/*give each LabChemTestSID and Sta3n a unique count #*/

/*get IQR on non-missing units dataset*/
PROC MEANS DATA=non_missing_unit_C4;
VAR LabChemResultNumericValue;
by Agg_count;
output out=non_missing_unit2(drop=_freq_) min= mean= median= std=  max= p10= p90=/ autoname;
RUN;

/*left join descriptives back to original dataset*/
PROC SQL;
	CREATE TABLE  non_missing_unit3  AS
	SELECT A.*, b.LabChemResultNumericVal_Median as Median, b.LabChemResultNumericValue_P10 as P10,
  b.LabChemResultNumericValue_P90 as P90
	FROM  non_missing_unit_C3  A
	LEFT JOIN  non_missing_unit2  B  ON A.Agg_count=B.Agg_count;
QUIT;


/*make a list of those uncertain LabChemTestSIDs, and then only create histograms for those uncertain ones*/
PROC SORT DATA=test;
BY  Labchemtestname  median;
RUN;

proc sgplot data=test noautolegend;
 histogram LabChemResultNumericValue;
 by Labchemtestname  median;
run;
```

Step 5: Create daily high and low values for labs

After labs are cleaned based on the above procedures, turn lab values into daily high and low values for each patient-day. This can be saved as a permanent dataset, so that it can be left joined to the VAPD by patient and day variables.

Example Code:

```
/*create HI & LO values by date*/
PROC SQL;
CREATE TABLE all_ALT_hi_lo_2014_2017 (compress=yes)  AS  /*17,031,334*/
SELECT *, max(LabChemResultNumericValue) as hi_ALT_daily, min(LabChemResultNumericValue) as lo_ALT_daily
FROM  alt2014_2017_20190409_v4
GROUP BY patienticn, LabSpecimenDate
ORDER BY patienticn, LabSpecimenDate;
QUIT;

PROC SORT DATA=all_ALT_hi_lo_2014_2017   nodupkey out=sepsis.ALT_hi_lo_20142017_20190417 ; /*16701006*/
BY  patienticn LabSpecimenDate hi_ALT_daily lo_ALT_daily;
RUN;
```

Step 6: Perform Spot Checks

- Data validation is done in VistAWeb or JLV. Request access if not granted already.
- Match verified labs to the cohort of interest (i.e., VAPD).
- Randomly select ~50 patient-facility-days on which a given lab was drawn and ~10 on which the lab was not drawn (in order to ensure that missing labs in CDW were not collected that day).
    - Because the final lab dataset doesn't include specific patient information that are needed to validate on VistAWeb/JLV, additional data processing is needed. On the validation list/file, include:
        - PatientSSN
        - DOB (from VAPD)
        - Facility
        - VISN #
        - DistrictName
        - City
        - Datevalue
        - Admit date
        - Discharge date
        - LabChemSpecimenDateTime
        - Lab variables to fill in the values
    - Have another person on the team (blindly) identify the lab values from that day from CPRS/VistA/JLV (or indicate that lab was not drawn that day), and compare to the extracted CDW lab values. A clinician can have a second look if anything is questionable.

Example Codes using SAS on vhaannapphsrd3:

1. Merge labs with VAPD or study population
2. Randomly select a cohort to validate
3. Get additional patient info on the cohort to validate, example code below

```
PROC SQL;
CREATE TABLE  cohort_crosswalk AS
SELECT distinct PatientSSN, PatientICN,PatientIEN, Sta3n
FROM Src.CohortCrosswalk;
QUIT;

DATA   cohort_crosswalk;
SET   cohort_crosswalk;
Patient_ID= input(patienticn, 10.);
RUN;

/*get SSN*/
PROC SQL;
        CREATE TABLE  vistaweb  AS
        SELECT A.*, B.PatientSSN
```

```
    FROM  work.validate_cohort A
    LEFT JOIN cohort_crosswalk B
    ON A.patienticn2=B.Patient_id  and a.sta3n=b.sta3n;
QUIT;

/* get VISN #*/
PROC SQL;
    CREATE TABLE vistaweb3 AS
    SELECT A.*, B.Facility, b.VISN, b.DistrictName, b.City
    FROM  vistaweb   A
    LEFT JOIN dim.vistasite  B
    ON A.Sta3n=B.Sta3n;
QUIT;
```

Use this SAS dataset or export SAS code to vistaweb3 into Excel or CSV format to keep the list in a secure folder.

For a complete list of lab extractions and cleaning codes, please refer to Github:

https://github.com/CCMRcodes/VAPD

Appendix C: Standard operating procedure for medications data extraction

Goal: Identify medications administered during an inpatient stay on a day-by-day basis. This SOP provides step-by-step instructions on how to extract pharmacy data from Corporate Data Warehouse (CDW). Medications of interest are extracted annually by calendar year.

Data Organization:

- CDW: Data stored in the Corporate Data Warehouse are organized as relational tables. Data are separated into multiple domains (such as vital signs, laboratory, inpatient, outpatient, etc) and tables within each domain. Linking keys (ending in 'SID') are used to reassemble data elements of interest to create tables for analysis.
- Dimension (Dim) tables: Supporting tables which hold meta data. For example, the Inpatient diagnosis tables would contain a key for a diagnosis code and the diagnosis dim table would provide the actual diagnosis code value.
- Pharmacy Bar Code Medication Administration (BCMA) Domain: Describes the medication administration process in the inpatient setting. Several types of information are available including the dates and time the medication was ordered, delivered and administered to the patient. Details of the medication (name, form, dose, routes of administration, additives and ingredients) are stored in this domain as well.
- PatientICN: Unique patient ID. Each facility has an ID for a patient (PatientSID) so that a patient seen at multiple facilities would have multiple PatientSIDs but the PatientICN is unique at the patient-level.

Step 1: List of Target Medications

- Principle Investigators generate a list of target medications

Step 2: Search and Identify All LocalDrugSIDs Associated with Medications of Interest

- Character search medications of interest from all fields containing drug names. For example, using LIKE operator can search for specific pattern or words in a column.
    - WHERE a.LocalDrugNameWithDose like '%PROPOFOL%' finds any values in LocalDrugNameWithDose field that have 'PROPOFOL' in any position.
- Extract the LocalDrugSIDs for all the matching medications from the Dim tables.
- There are three Dim tables containing inpatient medication administration:

A. <u>Dim.LocalDrug:</u>

Results Messages

	LocalDrugSID	Sta3n	LocalDrugNameWithDose	VAClassification	NationalDrugSID	DrugClassSID	DrugClass	DrugNameWithoutDoseSID	DrugNameWithoutDose
1	350	528	PROPOFOL 10MG/ML INJ 50ML	CN203	244679	8187	CN203	42658	PROPOFOL
2	686	528	PROPOFOL 10MG/ML INJ 20ML	CN203	244679	8187	CN203	42658	PROPOFOL
3	3936	528	PROPOFOL 10MG/ML INJ 100ML	CN203	244679	8187	CN203	42658	PROPOFOL
4	11531	528	ZZPROPOFOL 10 MG/ML INJ 50ML	CN203	244679	8187	CN203	42658	PROPOFOL
5	18548	503	PROPOFOL 10MG/ML, 20ML AMP	CN203	264865	933	CN203	83850	PROPOFOL
6	19779	506	PROPOFOL 10MG/ML INJ--50ML	CN203	281227	22257	CN203	60352	PROPOFOL
7	23622	506	PROPOFOL 10MG/ML INJ--100ML	CN203	281227	22257	CN203	60352	PROPOFOL
8	27055	506	PROPOFOL 10MG/ML INJ--20ML	CN203	281227	22257	CN203	60352	PROPOFOL
9	27423	637	ZZPROPOFOL 10MG/ML, 20ML AMP	CN203	301584	18923	CN203	48085	PROPOFOL
10	29602	508	PROPOFOL 10MG/ML, 50ML VIAL	CN203	312140	11773	CN203	76486	PROPOFOL
11	29603	508	PROPOFOL 10MG/ML, 100ML VIAL	CN203	312140	11773	CN203	76486	PROPOFOL
12	31451	637	PROPOFOL 10MG/ML EMULSION/...	CN203	301584	18923	CN203	48085	PROPOFOL
13	35869	508	PROPOFOL 10MG/ML, 20ML AMP/...	CN203	312140	11773	CN203	76486	PROPOFOL

```
/*get all the LocalDrugSIDs associated with list of drugs*/
/*first pull all LocalDrugSIDs*/
PROC SQL;
CREATE TABLE  localdrugsid  AS
SELECT a.DrugNameWithoutDose, a.LocalDrugNameWithDose,  a.NationalDrugNameWithDose,a.NationalDrug, a.Sta3n,
a.LocalDrugSID
FROM  Dim.LocalDrug AS A
WHERE  a.LocalDrugNameWithDose like '%PROPOFOL%' OR a.DrugNameWithoutDose like '%PROPOFOL%'
      OR a.NationalDrug like '%PROPOFOL%' OR a.NationalDrugNameWithDose like '%PROPOFOL%';
QUIT;
```

B. Dim.IVSolutionIngredient:

Results | Messages

	IVSolutionIngredientSID	Sta3n	IVSolutionFirstIngredientPrintName	LocalDrugSID	LocalDrugNameWithDose	Volume
1	800000090	436	PROPOFOL 1000MG/100ML	800150320	PROPOFOL 1000MG IN RTU 100ML	100 ML
2	800000129	442	PROPOFOL 1000MG/EMULSION	800082014	PROPOFOL 10MG/ML 100ML INJ	100 ML
3	800000130	442	PROPOFOL 500MG/EMULSION	800076476	PROPOFOL 10MG/ML (DIPRIVAN) 50ML INJ	50 ML
4	800000439	501	PROPOFOL 10MG/ML	800105301	PROPOFOL 10MG/ML INJ 50ML (VI)	50 ML
5	800000502	504	PROPOFOL	800142657	PROPOFOL 10MG/ML INJ, 100ML	100 ML
6	800000563	519	PROPOFOL 10MG/1ML EMULSION	800159387	PROPOFOL 10MG/ML INFUSION (50ML)	50 ML
7	800000588	519	PROPROL 1% EMULSION 100ML	800157032	PROPOFOL 1% EMULSION FOR INFUSION 100ML	100 ML
8	800000589	519	PROPOFOL 1% EMULSION 100ML	800157032	PROPOFOL 1% EMULSION FOR INFUSION 100ML	100 ML
9	800000598	519	PROPOFOL 10MG/ML	800212767	PROPOFOL 10MG/ML 20ML INFUSION	20 ML

```
/*[Dim].[IVSolutionIngredient]*/
PROC SQL;
CREATE TABLE  IVSolutionIngredient  AS
SELECT a.IVSolutionIngredientSID, a.LocalDrugNameWithDose, a.Sta3n, a.LocalDrugSID, a.Volume,
a.IVSolutionFirstIngredientPrintName
FROM  Dim.IVSolutionIngredient AS A
WHERE a.LocalDrugNameWithDose like '%PROPOFOL%' or a.IVSolutionFirstIngredientPrintName like '%PROPOFOL%';
QUIT;
```

C. Dim.IVAdditiveIngredient:

Results | Messages

	IVAdditiveIngredientSID	Sta3n	IVAdditiveIngredientPrintName	LocalDrugSID	LocalDrugNameWithDose	DrugUnit
1	800000152	504	PROPOFOL	800142657	PROPOFOL 10MG/ML INJ, 100ML	ML
2	800000583	600	z-PROPOFOL	800174587	PROPOFOL 10MG/ML 50ML VI	ML
3	800000867	653	PROPOFOL	800046532	PROPOFOL 10MG/ML INJ 20ML	MG
4	800001057	501	PROPOFOL	800109038	PROPOFOL 10MG/ML INJ 100ML (VI)	MG
5	800001646	691	PROPOFOL	800087744	PROPOFOL 10MG/ML INJ VIAL 20ML	MG
6	800002292	648	PROPOFOL**	800026064	PROPOFOL 10MG/ML INJ 100ML	MG
7	800002308	678	PROPOFOL	833780	PROPOFOL 10 MG/ML INJ. 50 ML RTU VIAL	MG
8	800004108	570	PROPOFOL	800066862	PROPOFOL 10MG/ML (20ML) INJ,EMULSION	MG

```
/*[Dim].[IVAdditiveIngredient]*/
PROC SQL;
CREATE TABLE  IVAdditiveIngredient  AS
SELECT a.IVAdditiveIngredientSID, a.LocalDrugNameWithDose,  a.Sta3n,  a.LocalDrugSID, a.DrugUnit,
a.IVAdditiveIngredientPrintName
FROM Dim.IVAdditiveIngredient AS A
WHERE  a.LocalDrugNameWithDose like '%PROPOFOL%' or  a.IVAdditiveIngredientPrintName like '%PROPOFOL%';
QUIT;
```

Step 3: Remove Duplicate LocalDrugSIDs, Screen Exclusions, & Create Drug_name Field

- Combine all data extractions from the three Dim tables, remove duplicate LocalDrugSIDs.
- Screen drug names to exclude any medications with word “research” and/or “study”.
- Label each LocalDrugSID as a medication indicator fields.

```
/*label LocalDrugSIDs with drug_name field*/
PROC SQL;
CREATE TABLE pharm3 AS
SELECT *,
             case when LocalDrugNameWithDose  like '%PROPOFOL%' or LocalDrugNameWithDose  like  'PROPOFOL%' or
            LocalDrugNameWithDose  like 'ZZ DIPRIVAN%' or LocalDrugNameWithDose  like 'DIPRIVAN%'  then 'PROPOFOL'
                        END AS drug_name
FROM all_undup_localdrugsids_table
QUIT;
```

Step 4: Extract Medication Administrations Data from Inpatient BCMA Tables

- To identify date and time for each medication administration to specific patients.

For each BCMA Table:

<u>Src.BCMA_BCMADispensedDrug:</u>

Results | Messages

	BCMADispensedDrugSID	BCMAMedicationLogSID	Sta3n	ActionDateTime	LocalDrugSID
634	1000000409547	1000000419095	556	[redacted]	662440
635	1000000409548	1000000419096	556		661935
636	1000000409548	1000000419096	556		661935
637	1000000409549	1000000419097	556		662445
638	1000000409549	1000000419097	556		662445
639	1000000409550	1000000419098	556		661889
640	1000000409550	1000000419098	556		661889
641	1000000409551	1000000419099	556		660393

- Only select the LocalDrugSIDs in step 3, after removing the duplicates.

```
/*pull BCMA_pharm tables*/
/*get 2014 BCMA_BCMADispensedDrug*/
PROC SQL;
create table BCMADispensedDrug as
SELECT a.*
FROM  Src.BCMA_BCMADispensedDrug as A
where a.ActionDateTime >= '2014-01-01' and a.ActionDateTime <='2014-12-31'
  and A.LocalDrugSID IN (SELECT LocalDrugSID FROM pharm3);
/*only select those localdrugsids*/
quit;

/*get drug_name field that was created*/
PROC SQL;
      CREATE TABLE  BCMADispensedDrug_3 AS
      SELECT A.*, B.drug_name
      FROM  BCMADispensedDrug   A
      LEFT JOIN  pharm3 B
      ON A.LocalDrugSID=B.LocalDrugSID;
QUIT;
```

Src.BCMA_BCMASolution:

	BCMASolutionSID	BCMAMedicationLogSID	Sta3n	ActionDateTime	IVSolutionIngredientSID
1	1200000219505	1200058143199	516	[redacted]	1200006225
2	1200000219505	1200058143199	516	[redacted]	1200006225
3	1200000219506	1200058143200	516	[redacted]	1200006226
4	1200000219506	1200058143200	516	[redacted]	1200006226
5	1200000219566	1200058143261	516	[redacted]	1200006225
6	1200000219566	1200058143261	516	[redacted]	1200006225
7	1200000219627	1200058143354	516	[redacted]	1200004714
8	1200000219705	1200058143466	516	[redacted]	1200006225

- Only select the IVSolutionIngredientSIDs in step 2B.
- Link IVSolutionIngredientsSIDs with LocalDrugSIDs to get drug_name field.

```
/*get BBCMA_BCMASolution*/
PROC SQL;
create table temp.BCMA_Solution  as
SELECT a.*
FROM  Src.BCMA_BCMASolution as A
where a.ActionDateTime >= '2014-01-01' and a.ActionDateTime <='2014-12-31' and IVSolutionIngredientSID IN (SELECT
IVSolutionIngredientSID FROM IVSolutionIngredient);
QUIT;

PROC SQL;  /*get LocalDrugSID*/
    CREATE TABLE BCMA_Solution3  AS
    SELECT A.*, b.LocalDrugSID
    FROM  BCMA_Solution    A
    LEFT JOIN  IVSolutionIngredient B
    ON A.IVSolutionIngredientSID=B.IVSolutionIngredientSID;
QUIT;

PROC SQL;  /*get drug_name*/
    CREATE TABLE BCMA_Solution4 AS
    SELECT A.*, b.drug_name
    FROM  BCMA_Solution3  A
    LEFT JOIN  pharm3 B
    ON A.LocalDrugSID=B.LocalDrugSID;
QUIT;
```

Src.BCMA_BCMAAdditive:

	BCMAAdditiveSID	BCMAMedicationLogSID	Sta3n	ActionDateTime	IVAdditiveIngredientSID
905	1200001087250	1200002702183	581	[redacted]	1200014828
906	1200001087250	1200002702183	581	[redacted]	1200014828
907	1200001087251	1200002702207	581	[redacted]	1200014828
908	1200001087251	1200002702207	581	[redacted]	1200014828
909	1200001087252	1200002702212	581	[redacted]	1200003109
910	1200001087252	1200002702212	581	[redacted]	1200003109
911	1200000225068	1200058171834	516	[redacted]	1200000393

- Only select the IVAdditiveIngredientsSIDs in step 2C.
- Link IVAdditiveIngredientsSIDs with LocalDrugSIDs to get drug_name field.

```
/*get BBCMA_BCMAAdditive*/
PROC SQL;
create table temp.BCMA_BCMAAdditive  as
SELECT a.*
FROM  Src.BCMA_BCMAAdditive as A
where a.ActionDateTime >= '2014-01-01' and a.ActionDateTime <='2014-12-31' and IVAdditiveIngredientSID IN (SELECT
IVAdditiveIngredientSID FROM  IVAdditiveIngredient);
QUIT;

PROC SQL;  /*get LocalDrugSID*/
	CREATE TABLE BCMA_BCMAAdditive3  AS
	SELECT A.*, b.LocalDrugSID
	FROM  BCMA_BCMAAdditive  A
	LEFT JOIN   IVAdditiveIngredient B
	ON A.IVAdditiveIngredientSID=B.IVAdditiveIngredientSID;
QUIT;

PROC SQL;  /*get drug_name*/
	CREATE TABLE BCMA_BCMAAdditive4  AS
	SELECT A.*, b.drug_name
	FROM  BCMA_BCMAAdditive3  A
	LEFT JOIN  pharm3 B
	ON A.LocalDrugSID=B.LocalDrugSID;
QUIT;
```

- Create action_date from ActionDateTime for each. EX: ActionDate=datepart(ActionDateTime);
- Get unique patient ID: patienticn.
- Combine the three datasets: BCMA_BCMAAdditive4, BCMA_Solution4, and BCMADispensedDrug_3.

Step 5: Remove Duplicates and Reformat Dataset

- Remove duplicates by unique patient, drug_name, and action_date.
- Transpose data so that each row is a patient-facility-day.

```
/*transpose dataset if needed*/
DATA trans_all_otherdrugs (compress=yes);
SET final.other_drugs;
keep patienticn ActionDate drug_name;
RUN;

PROC TRANSPOSE DATA=trans_all_otherdrugs OUT=final.trans_all_otherdrugs  (DROP=_NAME_)  PREFIX=drugname_;
BY patienticn ActionDate;
VAR  drug_name;
RUN;
```

Step 6: Spot Checks

- Randomly select ~50 patient-days of drug delivery and ~10 patient-days of non-delivery to validate in CPRS/VistA. A clinician can have a second look if there are discrepancies.

For a complete list of inpatient pharmacy extractions and cleaning codes, please refer to Github:

https://github.com/CCMRcodes/VAPD

Appendix D: Antibiotic drug classifications reference

Antibiotic_Route	Variable Name	Label (Abx Class)
Penicillin_IV	abx1	penicillin
Amoxicillin_PO	abx1	penicillin
Amoxicillin/Clavulanate_PO	abx1	penicillin
Amoxicillin/Clavulanate_IV	abx1	penicillin
Ticarcillin/Clavulanate_IV	abx1	penicillin
Ampicillin/Sulbactam_IV	abx1	penicillin
Ampicillin_IV	abx1	penicillin
Ampicillin_PO	abx1	penicillin
Nafcillin_IV	abx1	penicillin
Piperacillin_IV	abx1	penicillin
Penicillin_PO	abx1	penicillin
Dicloxacillin_IV	abx1	penicillin
Dicloxacillin_PO	abx1	penicillin
Oxacillin_IV	abx1	penicillin
Piperacillin/Tazobactam_IV	abx2	anti_pseudomonal_pcn
Cefazolin_IV	abx3	1st_gen_cephalosporin
Cephalexin_PO	abx3	1st_gen_cephalosporin
Cefadroxil_PO	abx3	1st_gen_cephalosporin
Cefoxitin_IV	abx4	2nd_gen_cephalosporin
Cefuroxime_IV	abx4	2nd_gen_cephalosporin
Cefuroxime_PO	abx4	2nd_gen_cephalosporin
Cefaclor_PO	abx4	2nd_gen_cephalosporin
Cefprozil_PO	abx4	2nd_gen_cephalosporin
Cefotetan_IV	abx4	2nd_gen_cephalosporin
Cefixime_PO	abx5	3rd_gen_cephalosporin
Ceftibuten_PO	abx5	3rd_gen_cephalosporin
Ceftriaxone_IV	abx5	3rd_gen_cephalosporin
Ceftazidime_IV	abx5	3rd_gen_cephalosporin
Cefdinir_PO	abx5	3rd_gen_cephalosporin
Cefotaxime_IV	abx5	3rd_gen_cephalosporin
Ceftazidime/Avibactam_IV	abx5	3rd_gen_cephalosporin
Cefpodoxime_PO	abx5	3rd_gen_cephalosporin
Cefepime_IV	abx6	4th_gen_cephalosporin
Ofloxacin_PO	abx7	fluoroquinolone
Ofloxacin_IV	abx7	fluoroquinolone
Ciprofloxacin_IV	abx7	fluoroquinolone
Ciprofloxacin_PO	abx7	fluoroquinolone
Levofloxacin_IV	abx7	fluoroquinolone
Levofloxacin_PO	abx7	fluoroquinolone
Moxifloxacin_PO	abx7	fluoroquinolone
Moxifloxacin_IV	abx7	fluoroquinolone
Norfloxacin_PO	abx7	fluoroquinolone

Telavancin_IV	abx8	Vancomycin_IV
Dalbavancin_IV	abx8	Vancomycin_IV
Oritavancin_IV	abx8	Vancomycin_IV
Vancomycin_IV	abx8	Vancomycin_IV

Vancomycin_PO	abx9	Vancomycin_PO
Fidaxomicin_PO	abx9	Vancomycin_PO
Fidaxomicin_IV	abx9	Vancomycin_PO

Acyclovir_IV	abx10	antiviral
Acyclovir_PO	abx10	antiviral
Peramivir_IV	abx10	antiviral
Ganciclovir_PO	abx10	antiviral
Foscarnet_IV	abx10	antiviral
Ganciclovir_IV	abx10	antiviral

Azithromycin_PO	abx11	macrolide
Azithromycin_IV	abx11	macrolide

Metronidazole_PO	abx12	flagyl
Metronidazole_IV	abx12	flagyl

Trimethoprim/Sulfamethoxazole_PO	abx13	sulfa
Sulfamethoxazole_IV	abx13	sulfa
Sulfadiazine_PO	abx13	sulfa
Trimethoprim_PO	abx13	sulfa
Tetracycline_PO	abx13	sulfa
Trimethoprim/Sulfamethoxazole_IV	abx13	sulfa

Fluconazole_PO	abx14	antifungal
Fluconazole_IV	abx14	antifungal
Micafungin_IV	abx14	antifungal
Voriconazole_PO	abx14	antifungal
Voriconazole_IV	abx14	antifungal
Posaconazole_IV	abx14	antifungal
Posaconazole_PO	abx14	antifungal
Itraconazole_IV	abx14	antifungal
Itraconazole_PO	abx14	antifungal
Amphotericin B_IV	abx14	antifungal
Amphotericin B_PO	abx14	antifungal
Caspofungin_IV	abx14	antifungal
Anidulafungin_IV	abx14	antifungal

Aztreonam_IV	abx15	Aztreonam_IV

Clindamycin_IV	abx16	clinda
Clindamycin_PO	abx16	clinda

Daptomycin_IV	abx17	big_abx
Tigecycline_IV	abx17	big_abx
Linezolid_IV	abx17	big_abx
Linezolid_PO	abx17	big_abx
Ceftaroline_IV	abx17	big_abx
Tedizolid_PO	abx17	big_abx
Tedizolid_IV	abx17	big_abx
Colistin (Colistimethate Sodium)_IV	abx17	big_abx
Colistin (Colistimethate Sodium)_PO	abx17	big_abx
Polymyxin B_IV	abx17	big_abx
Ceftaroline_IV	abx17	big_abx
Ceftolozane/Tazobactam_IV	abx17	big_abx
Quinupristin/Dalfopristin_IV	abx17	big_abx

Gentamicin_IV	abx18	aminoglycoside
Amikacin_IV	abx18	aminoglycoside
Streptomycin_IV	abx18	aminoglycoside
Tobramycin_PO	abx18	aminoglycoside
Tobramycin_IV	abx18	aminoglycoside

Doxycycline_PO	abx19	tetracycline
Doxycycline_IV	abx19	tetracycline
Minocycline_PO	abx19	tetracycline
Minocycline_IV	abx19	tetracycline

Nitrofurantoin_PO	abx20	other
Fosfomycin_PO	abx20	other
